# Adaptation of Questionnaire about Aggressive Beliefs and Attitudes in Spanish Adolescents

**DOI:** 10.3390/ijerph19095050

**Published:** 2022-04-21

**Authors:** Carlos Salavera, Pablo Usán, Alberto Quílez-Robres

**Affiliations:** Department of Psychology and Sociology, Faculty of Education, University of Zaragoza, 50009 Zaragoza, Spain; salavera@unizar.es

**Keywords:** beliefs, attitudes, aggression, validation, adolescents

## Abstract

Aggressive beliefs and attitudes are increasingly present in adolescents, and it can be argued that they are a prevalent feature of adolescence. Michel, Pace, Edun, Sawhney, and Thomas’s (2014) original thirty-item scale was later shortened to a more parsimonious eight-item scale (ABA-SF). This study addresses the adaptation and validation of the brief Aggressive Beliefs and Attitudes Scale to Spanish adolescents. The sample comprised a group of Spanish adolescents (*N =* 771, *M age* = 14.01 years). A total of two studies were undertaken: (1) the scale was translated into Spanish and its internal consistency, factorial structure and convergent validity were established; and (2) factorial analysis was undertaken to confirm the questionnaire. The results yielded high scores for internal consistency, reliability (α = 0.82; Ω = 0.83) and convergent validity. The examination of the underlying nomological network revealed links with positive and negative feelings, anxiety and aggression. According to the Exploratory Factorial Analysis (EFA), the aggregate variance of the factors in the scale was 65.814%, indicating that they can explain variations in aggression levels in adolescents. For its part, the Factorial Confirmatory Analysis (FCA) confirmed the match between the translation and the model, leading to a sustainable model composed by the three factors identified and eight items: χ^2^ (17) = 30.693; *p* < 0.001; χ^2^/gL = 1.805; CFI = 0.968; NFI = 0.837; TLI = 0.944; RMSEA = 0.060, IC del 95% (0.048–0.072). The short scale is easy to understand and quick to complete and is thus considered a useful instrument to assess aggression levels in adolescents.

## 1. Introduction

Insults, threats, physical and psychological aggression, humiliation, etc., are increasingly common among adolescents, both in real life and on social media. Aggression prevalence rates vary significantly from study to study [1]. For instance, a number of meta-analytical studies have argued for prevalence rates ranging from 4% to 36% [2,3]. In part, this can be associated with differences in the conceptualization of various aggressive behaviours; the instruments used; the design and analytical methodology of each study; the chosen threshold for what can be regarded as aggressive. However, all these studies emphasize the worrying prevalence of these attitudes among adolescents.

This is linked to an increase in high-risk practices among adolescents, for instance their participation in potentially harmful activities (both physically and mentally), including physical injuries and exposure to violent behaviour, unsafe sexual practices, and the consumption of tobacco, alcohol and illegal substances [4,5]. Although these practices are often sporadic, if they are not detected early, and if consolidated patterns of risky behaviours are not effectively monitored, their consequences may be severe.

This has led to an increasing interest in the study of aggressive attitudes that can affect their behaviour directly, and in the design of effective interventions to prevent them in adult life. In addition, it is likely that those that suffer or display these aggressive behaviours can affect others and, in consequence, their behaviour as well. There are different types of aggressiveness in young people: physical (blows, kicks, etc.); verbal (insults); facial (angry expressions on the face); indirect (towards objects of the affected person); and sexual (abuse, rape, etc.).

Most of the existing studies on the factors which affect aggressive behaviour in adolescents are lateral in nature, are result-focused (high risk practices in adolescence), or address proximal factors, that is, those that post-date early infancy (before 6 years of age, a critical period of development). Few studies have examined the link between aggression and aggressive beliefs and attitudes (risk factors) [6,7]. The above measuring instruments are CRT-A Conditional Reasoning Test Aggression [8] and the Angry Hostility Scale of the NEO Personality Inventory [9], which did not address issues such as aggressive beliefs and attitudes. This study aims to adapt a brief scale used to assess these aggressive beliefs and attitudes in adolescents, an issue which is still poorly understood. A thorough understanding of the factors related to these aggressive beliefs and attitudes could help prevent them and, therefore, reduce aggression prevalence rates in this age group [10,11].

Many studies have addressed aggressive conduct in adolescents and its associated factors. The role of moral withdrawal as one of the main predictors of aggressive behavior along with other issues such as bullying has been widely described. This would help researchers to understand the processes that leads young people to engage in aggressive behaviors [12,13]. As a rule, these studies examine the factors associated with a single conduct, or the association of several conducts and a single factor [4,5,14,15,16,17], but not the background for these conducts, that is, previous beliefs and attitudes.

Implicit and explicit cognition are important factors to understand how personality affects behaviour [18,19,20,21,22,23]. Implicit and explicit cognition are theoretically and functionally different, but they explain the structure of personality, including beliefs, attitudes, and behaviour. Specifically, implicit social cognitions refer to automatic, effortless (that is, subconscious) processes, while explicit social cognitions refer to introspected, controlled (that is, conscious) processes [24,25,26,27]. The basic premise is that aggressive persons see and understand aggressive attitudes as reasonable, in contrast with non-aggressive persons [28,29]. The literature has extensively described the role of moral withdrawal as one of the main predictors of aggressive behavior along with other issues such as bullying. Therefore, aggressive persons rely on implicit cognitive biases to justify their behaviour [30,31,32,33,34]. These biases are: (1) Hostile attribution: they tend to see harmful intention in the actions of others; (2) Potency: they tend to frame reasons in terms of strength versus weakness; (3) Retribution: they tend to give reprisals priority over reconciliation; (4) Victimization by powerful others: they tend to see themselves as victims of the powerful; (5) Derogation of targets: they try to present the target as deserving of the aggression; and (6) Social discounting: they tend to look at the socially different and antisocial behaviours to interpret and analyse relationships.

In addition, explicit social cognitions, in contrast with the implicit, occur at a conscious level, so they are easily accessible to introspection by means of direct methods, such as self-administered questionnaires. However, both, the implicit and the explicit, are needed to explain how personality affects behaviour, and can be used to predict aggression [25,35].

Despite theoretical advances, no scale existed to measure the relationship between explicit social cognitions and James’s [30] six aggressive biases, and the evaluation of these social cognitions was based on personality-focused questionnaires.

The aggressive beliefs and attitudes questionnaire has demonstrated its psychometric validity, in both its original [22] and short, eight-item formats [36]. However, to date this questionnaire has not been translated into other languages and used in different cultural contexts or with adolescents. Owing to its brevity and simplicity, it was decided to translate the scale into Spanish, with a view to provide an additional tool for Spanish-speaking scholars to study these variables.

The aims of this study are as follows: (1) to evaluate the possibility of translating the aggressive beliefs and attitudes questionnaire in its short format [36] into Spanish and to adapt it to Spanish adolescents; and (2) to analyse the validity of the questionnaire in this context. In the first phase of the study, the questionnaire was translated into Spanish, and its internal consistency, factorial structure and convergent validity were established. In the second phase, the confirmatory factorial analysis of the questionnaire was undertaken.

The only hypothesis of the study is that the aggressive beliefs and attitudes questionnaire is a valid instrument to measure this construct in Spanish adolescents.

## 2. Method

In order to select the sample, schools were contacted telephonically, and a list of participating schools was drafted. The principal investigator explained the main objective of the study to the participants when the questionnaires were delivered, emphasizing the importance of responding to all issues. Participants had 45 min to complete the questionnaires and the informed consent form. Participants were informed that the information would be treated anonymously and confidentially. The data were collected in October and November 2021.

The translation of the Aggressive Beliefs and Attitudes Scale into Spanish followed the standard process of reverse translation set out by Muñiz, Elosua & Hambleton [37], which comprises the following steps:

1. The original English version was translated into Spanish by two bilingual translators (native Spanish speakers), with experience in the translation of scientific literature in the field of psychology. The translations were compared and examined by the research team, and a first Spanish draft was agreed.
2. A psychologist with professional experience in English-speaking countries assessed the conceptual equivalence, clarity and naturalness of items and responses in this first version, and a second version was drafted with the necessary corrections.
3. The new version was circulated to psychologists working with adolescents for comment.
4. The second Spanish version was translated back into English by a bilingual translator (native English speaker).
5. A pilot test with 50 secondary school students was undertaken in order to test the clarity of the questionnaire, the response time and the suitability of the responses.
6. Based on these preliminary results, a third version of the scale was drafted and tested to assess its suitability to Spanish adolescents.

### 2.1. Participants

The sample comprised 771 secondary school students from Zaragoza (Spain)—361 men (49.42%) and 451 women (50.58%)—all of whom were volunteers (Table 1). The average age was 14.01 years (*s.d.* = 1.67). Parents and participants were asked to sign an informed consent form, and all the ethical considerations set out in the Declaration of Helsinki were met; the study was endorsed by the Ethics Committee of research group OPIICS (S46_20R), University of Zaragoza; all ethical criteria for research with human beings were met (volunteer participation; informed consent and right to information; protection of personal data and full confidentiality; no discrimination; gratuity; and, possibility to abandon the study at any point). The sample was found to be representative of the province of Zaragoza, with a 99% confidence level. The study was designed as an ex–post facto retrospective study [38]. Results were analysed anonymously.

### 2.2. Instruments

To evaluate the variables studied, the following instruments were administered: Aggressive Beliefs and Attitudes Questionnaire-Short Form (allowed us to evaluate aggressive beliefs and attitudes), SPANE positive and negative feelings scale (for the evaluation of feelings) and Zuckerman–Kuhlman Personality Questionnaire ZKPQ-50-CC (to assess personality). Respondents were asked to fill out a biographical information form and the survey to assess the variables studied. At this point, single method bias is regularly mentioned as a limitation of the survey design. However, this was mitigated by the limited number of items contained in the research questionnaire, and furthermore, the questions were worded in a clear and concise manner [39]. Furthermore, respondents were assured of their anonymity, this reassurance contributed to genuine responses [40]. Finally, standardized questionnaires were administered.

1. *Aggressive Beliefs and Attitudes Questionnaire-Short format* [36].

Explicit aggressive beliefs and attitudes were originally assessed through a 30-item scale developed and validated by Michel et al. [22], and later a shorter version was developed [36]. It assumes a multidimensional construct, constituted by three different but interrelated factors: (1) victimization; (2) derogation; and (3) retribution. It comprises eight items and responses are presented on a seven-point Likert scale. It is regarded as highly reliable (α = 0.80). In the present study α = 0.82 (Ω = 0.83).

2. *SPANE positive and negative feelings scale* [41].

The positive and negative feeling scales evaluate the feelings experienced by the participant in the four weeks prior to the survey. They comprise 12 items, six for positive affects (PA) and six for negative feelings (NA), in two Likert scales. Respondents must refer to their feelings at the time of filling in the questionnaire, and responses range from 0 (absence of emotion) to 5 (frequent presence of emotion). All the SPANE items score from 1 to 5, where 1 means ‘very rarely or never’ and 5 means ‘often or always’. Positive and negative scales are examined separately, owing to the partial independence of separability of both types of feeling. The aggregates can range from 6 to 30 (positive, SPANE-P), and from −6 to −30 (negative SPANE-N). The aggregate of both scales (SPANE-B) can range from 24 to −24, which reflects the affective balance. The scale yielded a reliability score of α = 0.89; Ω = 0.90 for positive feelings and α = 0.86; Ω = 0.88 for negative feelings.

3. *ZKPQ-50-CC* [42].

It is a self-administered questionnaire used to assess personality. It comprises 50 items with true/false answers. It assesses the different dimensions of personality, using alternative five as factors: (1) Impulsive sensation seeking: willingness to run risks for excitement and new experiences; (2) Neuroticism-anxiety: emotional instability, tension, concern, phobias and/or fears; (3) Aggression-Hostility: verbal aggression, rudeness, antisocial behaviour and rancour; (4) Activity: need for action, inability to stay idle; and (5) Sociability: number of friends and time spent with them, preference for company over solitude. The scale of Aggression-Hostility (Agg-Host) is a combination of willingness to express oneself aggressively and rudeness, disregard, antisocial behavior, revenge and malice, or from the other pole, willingness to be pleasant and cordial and have a friendly treatment with people. In terms of internal consistency, the ZKPQ-50-CC yields an α score between 0.78 and 0.82, and its convergence validity is regarded as adequate. In this study, α values for the different factors ranged from 0.77 to 0.83; and Ω values from 0.79 to 0.84.

### 2.3. Data Analysis

*Study 1.* Descriptive statistics were undertaken to establish the sociodemographic characteristics of the data: averages and standard deviations for the quantitative variables and percentages for the nominal variables. Reliability and validity were calculated in order to establish the questionnaire’s psychometric properties. In terms of reliability, Cronbach’s alpha [43] was calculated as a measure of internal consistency of the scale, for each item the corrected alpha of the relevant scales and subscales without the item was used. Concerning convergent validity, Pearson’s correlation coefficients between each item and the corresponding subscales were calculated, in order to test whether any correlation was in excess of 0.40, seeking to evaluate a possible bidirectional linear association between the different continuous variables, taken two by two. In addition, correlations between subscales and between subscales and aggregate scale scores were calculated. Validity was tested through factorial analysis, with initial factor extraction, following the principal component analysis with oblique rotation (Oblimin), a method of exploratory factorial analysis widely used in the elaboration of questionnaires. For the inclusion of items in the factors, factorial weights of 0.400 or more were considered. For the validity of the factorial model, the Kaiser–Meyer–Olkin index was calculated and Bartlett’s test of sphericity was undertaken.

*Study 2.* The following goodness of fit indicators were examined: chi-square (χ^2^); comparative fit index (CFI); Tucker–Lewis’s index (TLI); and Root Mean Square Error of Approximation (RMSEA) with a 90% confidence interval. Based on typical criteria, CFI and TLI values of 0.90 and 0.95 are regarded as an indication of good and excellent fit, respectively, while RMSEA values below 0.08 and 0.06 are considered indicative of acceptable and excellent fit, respectively.

## 3. Results

The results are divided into two groups, one for each phase of the study. The following results correspond to Study 1: construct validity; internal consistency analysis; convergent validity; and Exploratory Factorial Analysis (EFA); the results for Study 2 correspond to the Confirmatory Factorial Analysis (CFA).

### 3.1. Study 1

#### 3.1.1. Construct Validity

The aim of the study was to validate the Aggressive Beliefs and Attitudes Questionnaire, in its short format [36], with adolescents. The first step after the translation was to test the construct validity of the questionnaire.

The principal components analysis with Oblimin rotation method was chosen after the viability of the factorial analysis was established, with the following criteria: the correlation matrix yielded a large number of correlations (85.1%) with values in excess of 0.30 (determinant = 0.002), and Bartlett’s test of sphericity indicated that variables were not independent (Bartlett test = 483.126, *p* < 0.001). The Kaiser Meyer Adequacy (KMO) test yielded a value of 0.716, suggesting that the correlations between pairs of variables can almost be totally explained by the remaining variables. All Measures of Sampling Adequacy (MSA) values were above 0.75. These values recommended the correlation matrix to be subject to factorial analysis. As shown in Table 2, three factors with an eigenvalue over 1 were found, using as criterion the assignation to factors of items with a factorial weight over 0.40, which explained 65.81% of total variance. The greatest saturation value was associated with Factor 1, derogation (34.73%), followed by Factor 2, victimization (18.85%) and retribution (12.22%).

The matrix of components extracted through principal component analysis and the questionnaire items, and their saturation is presented in Table 3. For comparison purposes, it was decided to consider a goodness of fit if the reason between Chi-square and the degrees of freedom is below three [44]. By scale, their value was below three, which indicates their goodness of fit and internal consistency.

#### 3.1.2. Convergent Validity

Afterwards, the different components of the factors were analyzed to establish significant correlations between the dimensions of the questionnaire: victimization, derogation, and retribution. The resulting Pearson’s coefficients are presented in Table 4.

Correlations between the ABA-SF and the ZKPQ-50-CC and SPANE questionnaires were also examined (Table 5). Significant correlations with both instruments were attested. The victimization factor was found to only negatively correlate with anxiety (r = −0.123 *), that is, the greater the anxiety, the less victimization, and vice versa; derogation and retribution were also found to correlate negatively with anxiety (r = −0.271 **; r = −0.218 **), aggression-hostility (r = −0.285 **; r = −0.365 **) and positive feelings (r = −0.146 *; r= −0.132 *), and to correlate positively with negative feelings (r = 0.205 **; r = 0.132 *); that is, the higher the derogation and/or retribution values the greater the values that measure negative feelings.

#### 3.1.3. Reliability

In order to test the reliability of the adapted instrument, Cronbach’s alpha was used to establish internal consistency. All items returned an alpha value near or above 0.80 (Table 6), and it can, therefore, be assumed that the items that constitute them measure the same construct and are strongly correlated. Values of 0.8 or above are generally considered as indicative of good consistency, and the adapted questionnaire yielded a value of 0.82.

### 3.2. Study 2

Confirmatory factorial analysis was used to examine the internal structure of the scale; this is the adequate statistical framework to establish validity and reliability, not only globally, but also of each item, guiding and optimising the construction and adaptation of the questionnaire [45]. 

**Figure 1:**
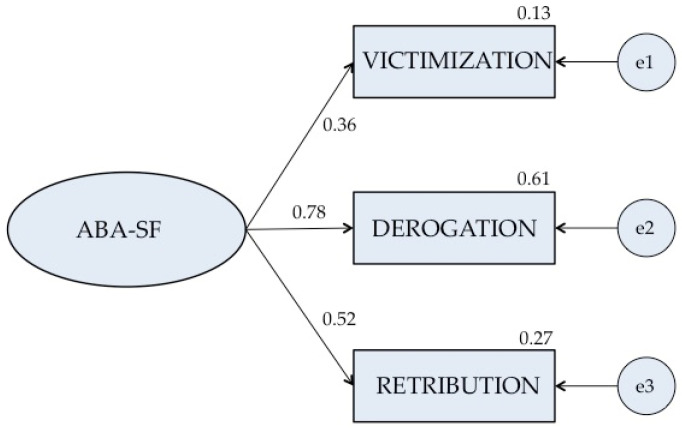
Standardized solution of the confirmatory factorial analysis, ABA-SF in adolescents.

Figure 1 illustrates the results of the Confirmatory Factorial Analysis (CFA) of the model generated by exploratory analysis, using structural equations (maximum likelihood method). The analysis returned a sustainable model of three factors and eight items, which confirms the adequacy of the model.

Concerning the goodness of fit, the various fitness indexes returned adequate values, suggesting that the proposed model for the factorial structure of the scale is sustainable: χ^2^(17) = 30.693; *p* < 0.001; χ^2^/gL = 1.805; CFI = 0.968; NFI = 0.837; TLI = 0.944; RMSEA = 0.060, IC del 95% (0.048–0.072).

The confirmatory factorial analysis model had three factors. This is an a priori structure, which means that the results of the model are entirely confirmatory. The indices returned a reasonably adequate fit; the χ^2^ value was 30.693 with 17 degrees of freedom; and RMSEA yielded an optimum fit, with an index of 0.060 (Lo = 0.048–Hi = 0.072). These results (Table 7) suggest that the model approaches the data optimally, and contributes to endorse the validity of the questionnaire. In this model, indices CFI, TLI and RFI returned values above 0.90 and the RMSEA was small (0.060), again suggesting an acceptable fit to the data. The fit of the data to a three-component hierarchical model (Table 7) was significantly better than that yielded by a unidimensional hierarchical model (χ^2^ = 69.798, gL = 20, *p* < 0.001).

## 4. Discussion

The aim of this study was to explore the use of the ABA-SF scale to measure aggression in adolescents.

The study validated the ABA-SF scale, following a protocol that included the translation, adaptation, and psychometric evaluation of the questionnaire. The article presents the protocol followed to ensure the cultural and linguistic equivalence of the original scale and the adapted version, and the tests undertaken to establish its internal validity and factorial structure.

The results of factorial analysis yield a three-dimension structure and high saturation in all items, which is a reflection of good internal consistency (0.851), similar to those returned by the questionnaire in earlier studies [22,36]. 

The results clearly showed that the factors of the ABA-SF scale present an accumulated variance of 65.81%. Other variables also support the validity of the questionnaire, such as the high consistency attested between elements of the scale, which is indicative of great reliability.

In addition to exploring the underlying factorial structure, reliability indices for each subscale, similar to those returned by the original model, were also established. It can thus be confirmed that the questionnaire keeps consistent when used with adolescents. The three-factor structure was confirmed with empirical data. The three underlying factors are significantly correlated: Victimization-Derogation (r = 0.771 **); Victimization-Retribution (r = 0.678 **); Retribution-Derogation (r = 0.403 **); all of them are above 0.4, and two of them can be regarded as very strong correlations.

In a similar way, the analysis of the correlations of the scale with those that assess personality and emotions reveals that victimization, which reflects the individual’s tendency to feel victimized by the powerful, only correlates with neuroticism-anxiety, which suggests that adolescents that feel victimized are less prone to anxiety, probably reflecting an inclination to surrender to adversity. The derogation factor, the aggressor presenting the victim as deserving of the aggression, correlates negatively with anxiety, aggression and positive feelings, and positively with negative feelings. Finally, retribution, which measures the tendency to give priority to reprisals over reconciliation, correlates negatively with anxiety, aggression and positive affects—anxiety, aggression and positive affects levels drop after the reprisal—and positively with negative feelings. Therefore, the study has found preliminary evidence for correlations between the ABA-SF, BFQ-NA (personality) and SPANE (feelings) questionnaires. Moreover, our results satisfactorily responded to the underlying theoretical model and returned high internal consistency and validity values. As noted, social cognitions, both implicit and explicit, are important to understand the influence of personality on behaviour, and the ties of these constructs with social skills, self-esteem, emotions, sentiments, etc. [46,47,48,49,50] and even with psychopathologies that often emerge during adolescence, such as depression, mood swings, ADHD, etc. [51,52,53,54,55,56]. Therefore, future research must explore the relationship between the ABA scale and these variables, and, going further, even re-examine its features.

These results must be interpreted in light of the limitations of the study. Although the sample is statistically significant, it should be expanded to include other population segments, in which different correlations between aggressive beliefs and attitudes and other constructs may be attested. It may also be accurate to study the difference between Spanish-speaking countries in terms of their conception of violence within their culture. It is also advisable to undertake longitudinal studies that cover the evolution of these constructs over a longer time period. In addition, aggressive behaviour is often compounded with family, school and social contextual factors, and this relationship should be explored further. The questionnaire should also be used to analyze population groups with special characteristics, such as adolescents suffering from anxiety or autism, which present a different profile in terms of social cognitions, both implicit and explicit [52,57,58]. 

The main conclusion of the study is that the ABA-SF is a valuable tool to measure aggressive beliefs and attitudes in adolescents; it is psychometrically efficacious, its factorial structure is solid, and it is internally consistent; in addition, it is quick and easy to implement. Although further research is necessary, these preliminary results indicate that the ABA-SF questionnaire is a useful tool to measure aggression in adolescents.

## 5. Conclusions

The results of the research endorses the validity of the ABA-SF as a short, easy to implement and understand, and adequate for the measurement of the target constructs, research tool. Besides, the factorial structure model proposed is sustainable; three factors and eight items were identified. The translated ABA-SF yielded satisfactory coefficients for each of the three factors identified in its factorial structure. In our perspective, this further demonstrates the validity of the scale in adolescents, and confirms that the Spanish version faithfully replicates the theoretical structure of the original.

## Figures and Tables

**Table 1:** Distribution of the sample by age (*N* = 771).

Age	Frequency	Percentage	Age	Frequency	Percentage
12	120	15.56%	15	193	25.03%
13	191	24.77%	16	58	7.52%
14	195	25.29%	17	14	2.46%

**Table 2:** Percentage of the variance explained by ABA-SF scale factors.

Factor	Total	% Variance Explained	% Accumulated Variance
Derogation	2.779	34.734	34.734
Victimization	1.509	18.858	53.592
Retribution	0.978	12.222	65.814

**Table 3:** Principal components matrix.

Item	Victimization	Derogation	Retribution
1	0.865		
2		0.835	
3	0.796		
4			0.954
5	0.792		
6			0.753
7			0.883
8		0.807	

**Table 4:** Pearson’s correlation coefficients in the ABA-SF scale.

	Victimization	Derogation
Victimization	1	
Derogation	0.771 **	1
Retribution	0.678 **	0.403 **

** *p* < 0.01.

**Table 5:** Correlations with personality and emotional factors.

	Victimization	Derogation	Retribution
Neuroticism-anxiety	−0.123 *	−0.271 **	−0.218 **
Impulsive sensation-seeking	−0.105	−0.052	−0.093
Activity	0.100	0.094	0.087
Sociability	0.010	0.120	0.088
Aggression-hostility	−0.041	−.285 **	−0.365 **
Positive feelings	−0.017	−0.146 *	−0.124 *
Negative feelings	−0.055	0.205 **	0.132 *

* *p* < 0.05, ** *p* < 0.01.

**Table 6:** Internal consistency of the ABA-SF factors.

Items	Average	DT	Correlation Item-Test	If Item Is Removed	Scale
Item 1	4.923	1.705	0.878 **	0.753	
2	5.308	1.702	0.517 **	0.803	Average = 5.053 Alpha = 0.820
3	5.600	1.779	0.629 **	0.786	
4	6.231	0.920	0.524 **	0.824	
5	4.615	1.325	0.517 **	0.802	
6	5.385	1.192	0.394 **	0.804	
7	3.154	1.281	0.736 **	0.839	
8	5.214	1.957	0.411 **	0.767	

** *p* < 0.01.

**Table 7:** Factorial confirmatory analysis of ABA-SF scale.

Model	χ^2^	gL	CFI	TLI	NFI	RMSEA
Three-factor model	30.393	17	0.968	0.944	0.937	0.060
One-factor model	69.798	20	0.884	0.796	0.857	0.114

## Data Availability

The data presented in this study are available on request from the corresponding author.
